# Retirement preparation program: evaluation of results

**DOI:** 10.1186/s41155-017-0079-3

**Published:** 2017-12-08

**Authors:** Tanise Amália Pazzim, Angela Helena Marin

**Affiliations:** Universidade do Vale do Rio dos Sinos (Unisinos), Unisinos Ave - E01-109, São Leopoldo, Zip code: 93.022-750 Brazil

**Keywords:** Retirement, Meaning of work, Quality of life

## Abstract

The present study aimed to evaluate the results obtained in the retirement preparation program **(**RPP) regarding changes in retirement planning behaviors, the meaning of work, and improvement in quality of life. It is a quasi-experimental research, using a pre- and post-test evaluation with a non-equivalent control group, including 82 participants, who were public workers near retirement. Among these, 50 participated in the RPP (experimental group (EG)) and 32 belonged to the control group (CG). All responded to the scale of changes in retirement planning behaviors, the Meaningful Work Scale, and the World Health Organization Quality of Life Assessment Instrument (WHOQOL-*Brief*), as well as a Sociodemographic and Occupational Data Questionnaire. The results showed that in the EG, there was an increase in the socio-occupational investment and in the coherence and expressiveness at work throughout the three periods considered, as well as in the quality of life related to the environment in the assessment conducted after the program. Regarding the comparison between the EG and the CG, differences were verified in the socio-occupational investment and in the social utility of work, and such characteristic was higher in the EG during the follow-up. The relevance of the present study lies in the improvement of intervention alternatives to support health policies, seeking to meet the demands of the aging population.

## Background

The onset of retirement life is an experience of great transformations and changes. The retired status may have negative and/or positive effects in the worker’s life. Positive factors are usually associated to the planning of this new phase, which may favor quality of life after retirement from work, maintenance of physical and mental health, and engagement in leisure activities or new formal or voluntary work (Wang et al. 2011). Retirement can also be felt as liberating due to the opportunity to complete previously unrealized plans (França 2008), enabling the planning of future projects (Costa and Soares 2009). Among the negative factors, national studies indicated depression and social isolation as motivators of suicide among Brazilian elders (Cavalcante and Minayo 2012; Minayoe et al. 2012), besides socioeconomic decline and financial exploitation of family members (Minayo et al. 2012). There is evidence that alcohol consumption can also increase in retirement, especially when it occurs involuntarily (Kuerbis and Sacco 2012). Therefore, it is verified that this moment is permeated by several feelings, sometimes ambivalent, which can lead to apprehension, anxiety, doubts, mood swings, psychosomatic illnesses, and diverse fears, such as the one of losing the status of “active employee.”

In order to make the work-retirement transition more smoothly, it is essential that retirement preparation programs (RPPs) be proposed in organizations (França and Soares 2009; Zanelli et al. 2010). Such programs are characterized by a set of actions and activities for people in the near-retirement phase, whom are those retiring within 2 to 5 years. This preparation is aimed at assisting people to prepare for the future, not only addressing financial aspects but also including a biopsychosocial vision of the human being, embracing the complexity of this moment (Pazzim et al. 2016).

Despite their relevance, the actions directed to retirement planning are still poorly assessed in Brazil (Murta et al. 2014b; Murta et al. 2014a; Nunes 2015). The reasons for the absence of a systematic evaluation may be related to the lack of knowledge of the evaluation methods or to the belief that such undertaking would be infeasible, complex, or costly (Murta et al. 2014b).

The evaluation process of RPPs must comprise five phases: needs assessment, development of programs, efficacy, effectiveness, and dissemination studies (Murta et al. 2014b). The *needs assessment* is the first step in the development of a retirement preparation program, and it aims to know the reality of work for the identification of the targets to be cared for (Murta et al. 2014b). The second step, *development*, consists in the creation of procedures and materials, the establishment of methods, and in the assessment of their acceptability by the participants (Murta et al. 2014a). The studies on the development of retirement education programs are monitored by process evaluations, also called formative assessments or monitoring assessments. The third step, called *efficacy assessment*, aims to investigate the quality of programs. When assessing the efficacy of an intervention or program, it is verified if the achievement of the goals proposed in the actions developed was caused by the strategies adopted (Flay et al. 2005; Murta et al. 2014b), and it is recommended that researchers use at least one control condition (Flay et al. 2005) related to the comparison of results with groups of people who did not participate in the intervention or who partially participated. The fourth stage, *effectiveness assessment*, refers to the changes and reach resulting from the intervention in the real-life environment, and it aims to verify the effects of the implementation of the program in the natural environment when conducted by the people or teams that are not specialists and/or researchers (Abreu 2012; Flay et al. 2005), but multiplication agents or professionals trained by the developers of the intervention, already submitted to efficacy assessment studies. Finally, the last step in the assessment of programs refers to the diffusion, consisting in the transfer of a program with evidence of good results from studies that meet the efficacy and effectiveness criteria for new groups, institutions, and communities.

Based on the above statements, the objective of the present study was to evaluate the results, especially the efficacy, obtained in a RPP carried out in a municipal public institution, regarding the outcomes: (a) change in retirement planning behaviors, (b) change regarding the meaning of work, and (c) quality-of-life improvement, which are considered important constructs in the process of retirement, comparing these results between a group of public workers who participated in the RPP and a group who did not participate, at the end of the program and 2 months later.

## Method

### Design

This is a quasi-experimental study, using a pre- and post-test design with a non-equivalent control group (Cozby 2009), and a quantitative and longitudinal approach. This design considered an experimental group (EG) (who participated in the program) and a non-equivalent control group (CG) (who did not participate in the program). Both groups were assessed before the program (T1) and were monitored after 2 months (T3–follow-up). The EG also responded the assessment tools after the termination of the program (T2).

The fact that the follow-up was carried out 2 months after the intervention was due to the profile of most participants, whose retirement was expected for the current year, as well as the right to vacation and bonus leaves.

### Participants

Participants were 82 workers of a municipal public institution in a major city of south of Brazil, who were near retirement (projected for the next 5 years). Among these, 50 people composed the EG and 32, the CG. The EG included workers who voluntarily enrolled in two editions of the program that occurred in 2016. A limit of 50 workers was established for the EG because in the last editions of the program, there was a mean of approximately 25 participants who completed the research for each group with 30 vacancies.^1^ All those who participated in the program agreed to participate.

The EG was mostly composed of women (78%), with mean age of 55.6 years (SD = 5.3), and 56% were married/civil union and 84% had children. Regarding education, there was a predominance of participants with post-graduation (48%) and with incomplete/complete higher education (34%). Only 18% had incomplete/complete secondary education. The positions were also quite diverse, with predominance of administrative assistant (28%), professor (16%), and nurse (8%), and were linked to several secretariats, mainly from health (28%), education (20%), and the sanitation department (16%). Regarding the length of service in the institution, most had completed between 21 and 30 years of service (42%). For 30% of participants, the projected retirement date was 2017 and for 26% of them, the actual year of the intervention (2016). It is noteworthy that for the EG, there were 50 participants in T1, 45 participants in T2, and 33 participants in T3.

The CG, in turn, was composed by other 32 workers who were selected according to the following criteria: they did not have the opportunity to participate in the RPP because there were no vacancies left, which was documented by a waiting list organized by the Department of Human Resources (*n* = 5); they enrolled in the course but dropped out before the start of the program (*n* = 4); selection is by convenience according to the time of 5 years for retirement (*n* = 23).

Most CG participants were women (65.6%), with mean age of 56.6 years (SD = 4.7), 53% were married/civil union, and 93.8% had children. In relation to education, most had complete/incomplete higher education, (40.7%), including 31.3% post graduated, 18.8% presenting complete/incomplete secondary education, and 9.4% complete/incomplete elementary school. The positions were also quite diverse, with predominance of administrative assistant (28%) and administrator (18.7%), linked to several Secretariats, most often from Strategic Planning and Budget (34.4%), Administration (28%) and Health (15.6%). The period of service at the institution was concentrated between 11 and 20 years (25%), 21 to 30 years (40.6%), 31 to 35 years (21.9%), and more than 25 years (12.5%). For 3.1% of participants, the retirement start date was projected to 2016; for 43.8% to 2017, for 28.1%, 2018, for 3.1%, 2019, and for 15.6% of participants, 2020. It should be noted that for the CG in T1, there were 32 participants and in T3, 31 public workers.

### Retirement preparation program

RPP was based on the theory of continuity, part of the theory of psychosocial aging, which understands that retirement is a transition that begins before the worker shutdown and continues until some years after the event itself. It rejects the lack of function of retirees and emphasizes the importance of voluntary work, paid or replaced by other activities that bring them satisfaction (França and Soares 2009). This program consists of nine 4-h meetings, held twice a week, for a 36-h course load, as recommended by the literature (Leandro-França 2016; Zanelli et al. 2010), which indicates that long-term programs be conducted in around 8 to 20 meetings, with a minimum of weekly, bi-weekly, or monthly frequency, and a total of approximately 40 h. A multidisciplinary team of educators, who are employed in the institution, holds the meetings from the fields of psychology, philosophy, social service, accounting, medicine, nutrition, physical education, administration, and journalism, that are oriented towards thematic that should work and how to organize the meeting by the first author of this study. The dynamics of each meeting consists in the presentation of the themes, conceptual exposition, and discussion, using various strategies of group mobilization (group techniques, presentation of videos, and exercises) so that participants experience the content presented. Table 1 presents the RPP content.

**Table 1:** Content of the RPP

No.	Themes	Program content	HL
1	Presentation of the RPP	Opening with RPP coordination representative Presentation of the RPP (objectives, purpose, and program content) Presentation of participants Survey of expectations Operational combinations	2 h
	Work world and meanings of retirement	Socio-historical construction of work Work and formation of social identity Senses and meanings of work Pleasure and suffering at work Meanings connected to retirement: stereotypes and prejudices Idleness and free time	2 h
2	Social security aspects	Social security: concept, general regime, and special welfare regime Procedures to apply for retirement Benefits and advantages of retirement Registration of contribution time Application and processing of the administrative process of retirement	4 h
3	Personal and professional planning	Personal and professional planning Post-career	4 h
4	Psychological and family aspects	Work and subjectivity Building of personal and professional identity Expectations and feelings about retirement The importance of interpersonal relationships and the construction of networks The family’s role in retirement Divorce, depression, anxiety, suicide, and use and abuse of alcohol and drugs	4 h
5	The practice of physical activity	Retirement as a moment of resumption of physical activity Physical conditioning Choosing the physical activity you like the most	4 h
6	Dietary reeducation	Retirement and aging Eating habits throughout life Consequences of a poor diet on health Dietary reeducation Nutrients and food groups Daily menu planning and organization	4 h
7	Health care in retirement	Definition of health Health care Primary, secondary, and tertiary prevention Risk and protection factors Prevalent diseases in the elderly population Indications of periodic examinations after the age of 50	2 h
	Interview with retirees	Interview conducted by a journalist with two retirees, usually a man and a woman, one with a higher education and another with high school or technical level	2 h
8	Financial planning in retirement	Revenue, expenses, funding, investment and indebtedness Fixed, optional, and extra costs Family budget planning and model suggestions	4 h
9	Life plan	What is a life plan? How to make a life plan? Models of plans Plan maintenance	2 h
	Assessment and Closure	Assessment of the program Closure––socializing	2 h

### Instruments

#### Questionnaire on sociodemographic and occupational data

This instrument aims to collect information regarding the identification of the participants (age, education, marital status, family configuration, position, time of service, and projected retirement year). The design of the instrument considered the sociodemographic data outlined by IBGE, and the occupational data were selected according to the models presented in the literature (Soares and Costa 2011; Zanelli et al. 2010). The RPP coordination team has used this questionnaire.

#### Scale of change in retirement planning behavior (SCRPB) (Leandro-França et al. 2014)

This instrument aims to understand how people progress towards the adoption and maintenance of behaviors that can improve their health conditions. The scale was constructed considering behaviors that are favorable to the adaptation and to retirement, identified in the literature review (Leandro-França 2012). The SCRPB presents two factors, factor 1 (occupational-social investment) and factor 2 (investment in autonomy and well-being). Factor 1 includes eight items distributed as follows: participating in community groups, investing in projects that can be adapted/implemented from retirement, taking continuous improvement courses in another area aimed at a second career, performing volunteer work in the community, dedicating to spiritual or religious practices, having a hobby, taking continuous improvement courses in the area of practice, and nurturing friendships. Factor 2 includes seven items: the regular practice of physical activities, investing time in family life, having financial investments for the future, practicing leisure activities, investing in the relationship with partner, having a healthier diet, scheduling appointments, and medical check-ups. It should be emphasized that the instrument presented satisfactory psychometric characteristics for the current sample (Fayers and Machin 2007). The Cronbach alphas obtained were .71 for factor 1 and .79 for factor 2.

#### Scale of meaningful work (SMW) (Bendassolli et al. 2015)

This instrument aims to verify if the work has meaning factors, i.e., each item/factor of this scale evaluates how much the person believes that their current work is close to what they expect it should be to be meaningful. It is expected, therefore, that the larger the factorial mean of each dimension of the construct, the greater the perception of meaning in work. The results should be determined considering each of the six factors that compose it and their respective items: social utility (utility of work to society; *α* = .77), ethics (existence of justice and fairness at work; *α* = .94), freedom (possibility of judgment to solve problems and to make decisions; *α* = .87), learning and development (achievement of objectives; *α* = .78), quality of relationships (interesting contacts and peer support; *α* = .73), and coherence and expressiveness (work corresponds to competencies and professional interests, allowing goals to be achieved and people to be heard; *α* = .87). The SMW was translated to Portuguese and validated in Brazil. The consistency indexes obtained (Cronbach alphas) were considered satisfactory to the current sample.

#### World Health Organization Quality of Life Assessment Instrument (WHOQOL-Brief) (The Whoqol Group 1998, adapted by Fleck et al. 2000)

This instrument consists of 26 questions, of which two are questions for global assessment of quality of life, and the others represent 24 facets that assess four specific domains of quality of life. The physical domain evaluates pain and discomfort, energy and fatigue, sleep and rest, mobility, activity in everyday life, drug dependence and treatments, and capacity for work (*α* = .77). The psychological domain evaluates positive feelings: thinking, learning, memory and concentration; self-esteem; body image and appearance; negative feelings; and spirituality/religion/personal beliefs (*α* = .80). The domain of social relations evaluates the aspects involved in personal relationships, social support, and sexual activity (*α* = .74). The environmental domain assesses physical security and protection, home environment, financial resources, health and social care, opportunity to acquire new information and skills, participation in recreation/leisure opportunities, physical environment (pollution/noise/traffic/climate), and transport (*α* = .76). The instrument has satisfactory characteristics of internal consistency, discriminant validity, criterion validity, concurrent validity, and test-retest reliability.

### Ethical and data collection procedures

Initially, contact was made with the Administration Secretariat, which is responsible for the implementation of the RPP at the institution contacted, to request the consent. After authorization, the project was submitted and approved by the Research Ethics Committee of Universidade do Vale dos Sinos, according to Resolution no. 466/12 of the National Health Council, which regulates research with human beings (CAEE 51408715.3.0000.5344).

The first author of this study, who also followed the development of the intervention, conducted all data collection. In the first day of the program, the objectives and procedures of this research were presented to the EG. The CG participants, accessed as previously described, were contacted individually for scheduling. We made it clear that they could obtain any clarification whenever wished, to decide freely on their participation. The Free and Informed Consent Form (TCLE) was signed, ensuring the volunteer privacy and confidentiality of the data, and they responded the research instruments.

After 2 months of the end of the program, participants from the EG and the CG were invited for a lecture with SEBRAE on the topic “Senior Entrepreneurship,” and the follow-up was conducted at this occasion. For those who were not able to participate in the event, contact was made for individual scheduling or questionnaires were sent by e-mail.

### Data analysis procedures

The closed questions of the SCRPB, WHOQOL-Brief, and SMW Sociodemographic and Occupational Data Questionnaire were quantitatively analyzed, considering their absolute and relative distributions, through descriptive and inferential statistics, as well as the measures of central tendency and variability, with the study of normality using the Shappiro Wilk test. In addition, the reliability (Cronbach’s alpha) was calculated for the current sample, and the coefficient was presented in parentheses at the “Instruments” section. To compare the scores between the assessments in T1 and T2, and the assessments in T1 and T3 of the EG, Student’s *t* test was used for paired data. For the comparison between EG and CG in T1 and T3, Student’s *t* test for independent samples was performed.

To compare the means assessed over the three times for the EG (T1, T2, and T3), the variance analysis for repeated measurements (one-way ANOVA) and Bonferroni’s post hoc test were used. The adequacy of the model was assessed by the homogeneity of variances and co-variances using the Levene test and sphericity using the Mauchly test. The magnitude of intragroup differences was calculated from effect size. An effect size value of .20 to .49 is considered small, while .50 to .79 is a moderate effect and ≥ .80, a large magnitude effect (Cohen 1988). The data were analyzed in the Statistical Package for Social Sciences program, version 20.0, and for the statistical decision criteria, significance was established at 5%.

## Results

The results were organized in two stages. The first stage included the comparisons between the evaluated times (T1, T2, and T3), considering only the EG. The second stage consists in the evaluation and comparison between the EG and CG in T1 and T3.

### Stage 1: comparisons between times assessed in the EG

Table 2 shows that considering the EG analysis that compared the means between the T1, T2, and T3 assessments, there was a significant difference in the SCRPB regarding the socio-occupational investment (*p* < .003), indicating a significant increase of the mean over time, i.e., the mean in T3 (3.79 ± 0.62) was significantly higher than the mean in T2 (3.20 ± 1.03), which, in turn, was higher than the mean in T1 (2.86 ± 1.04). For the SMW, only the factor coherence and expressivity at work (*p* < .002) presented higher mean values in T3 (5.01 ± 1.01). For the WHOQOL-Brief, the only significant difference occurred in the environmental domain (*p* < .04), for which the mean in T2 (3.91 ± 0.56) was significantly higher than the mean values in T1 (3.74 ± 0.59) and T3 (3.78 ± 0.61). The perception of quality of life by the majority of participants in T1 was identified as good (54%), very good (24%), and neither bad nor good (22%). In T2, the proportions were maintained (good 53.8%, very good 25.6%, and neither bad nor good 20.5%). In T3, the distribution was slightly different (good 57.6%, very good 24.2%, neither bad nor good 15.2%, and bad 3%). As to how satisfied they were with their health, there was not much variation between T1 and T2 either (T1; satisfied 57.1%, neither satisfied nor dissatisfied 18.4%, very satisfied 12.2%, neither satisfied nor dissatisfied 21.1%, very satisfied 18.4%, and dissatisfied 13.2%). There were more distinct perceptions in T3; satisfied (51.5%), very satisfied (27.3%), neither satisfied nor dissatisfied (12.1%), and dissatisfied (9.1%).

**Table 2:** Mean and standard deviation for the instruments SCRPB, SMW, and WHOQOL-Brief, according to the assessments in T1, T2, and T3 for the EG

Instruments and dimensions	Period of assessment	*n*	Mean	Standard deviation	*p* ^C^
SCRPB––socio-occupational investment	T1	21	2.86	1.04	.003
	T2		3.20	1.03	
	T3		3.79	0.62	
SCRPB––investment in autonomy and well-being	T1	23	4.68	0.94	.502
	T2		4.58	1.02	
	T3		4.53	0.87	
SMW––social utility	T1	25	5.58	0.67	.921
	T2		5.63	0.44	
	T3		5.60	0.56	
SMW––ethics at work	T1	26	4.43	1.02	.595
	T2		4.41	1.27	
	T3		4.54	1.08	
SMW––freedom at work	T1	28	5.03	0.91	.446
	T2		4.86	0.88	
	T3		4.89	0.69	
SMW––learning and development at work	T1	27	4.88	1.00	.578
	T2		5.03	0.87	
	T3		5.00	0.90	
SMW––quality of relationships at work	T1	26	4.97	0.98	.096
	T2		4.92	0.89	
	T3		5.19	0.72	
SMW––coherence and expressiveness at work	T1	24	4.35	0.95	.002
	T2		5.00	0.94	
	T3		5.01	1.01	
WHOQOL––physical domain	T1	26	3.96	0.65	.388
	T2		4.03	0.59	
	T3		3.89	0.55	
WHOQOL––psychological domain	T1	23	3.94	0.68	.449
	T2		4.04	0.69	
	T3		3.93	0.61	
WHOQOL––social domain	T1	25	3.69	0.78	.539
	T2		3.84	0.79	
	T3		3.77	0.63	
WHOQOL––environment domain	T1	24	3.74	0.59	.043
	T2		3.91	0.56	
	T3		3.78	0.61	

^C^Analysis of variance for repeated measures (one-way)––Bonferroni’s post hoc test

### Stage 2: assessment and comparison of the EG and the CG

The instruments investigated in this study had their information compared between the EG and the CG. According to the results presented in Table 3, in the SCRPB, there was no significant difference (*p* < .44) between the groups for the social-occupational investment in T1; however, in T3, the average in the EG (3.74 ± 0.58) was significantly higher than in the CG (3.11 ± 0.93, *p* < .002). Similar results occurred in the SMW for the item social utility, in which the means in T1 did not differ representatively (*p* < .143); however, in T3, a difference was verified (EG 5.63 ± 0.53 vs. CG 4.93 ± 1.17, *p* < .005). Also, in the SMW, the item freedom at work presented significant difference, indicating a higher mean for the EG both in T1 (EG 4.79 ± 1.02 vs. CG 4.13 ± 1.35; *p* = .01) and in T3 (EG 4.91 ± 0.71 vs. CG 4.26 ± 1.24; *p* = .01). In the WHOQOL-Brief, when comparing the variations observed between the EG and CG, the means were not representative in this sample. The perception of quality of life by the majority of the CG participants in T1 and T2 did not appear to be different (T1; good 65.6%, very good 21.9%, and neither bad nor good 12.5%. T3; good 66.7%, very good 20%, neither bad nor good 10%, and bad 3.3%). As for health satisfaction, the same trend was observed (T1; satisfied 50%, very satisfied 25%, neither satisfied nor dissatisfied 12.5%, dissatisfied 9.4%, and very dissatisfied 3.1%. T3; satisfied 53.3%, very satisfied 23.3%, neither satisfied nor dissatisfied 13.3%, and dissatisfied 10%).

**Table 3:** Mean and standard deviation for scores of the instruments SCRPB, SMW, and WHOQOL-Brief, according to the assessments T1 and T3 on the total of valid cases

Instruments and dimensions	Period of assessment	Groups						
		Experimental––EG			Control––CG			*p* ^B^
		*n*	Mean	Standard deviation	*n*	Mean	Standard deviation	
SCRPB––socio-occupational investment	T1	40	3.15	1.10	32	2.94	1.18	.442
	T3	32	3.74	0.58	30	3.11	0.93	.002
SCRPB––investment in autonomy and well-being	T1	43	4.67	0.91	32	4.58	1.03	.692
	T3	32	4.68	0.85	30	4.63	1.11	.842
SMW––social utility	T1	50	5.43	0.89	32	5.10	1.11	.143
	T3	32	5.63	0.53	30	4.93	1.17	.005
SMW––ethics at work	T1	50	4.40	1.26	32	4.36	1.40	.512
	T3	34	4.56	1.14	30	4.40	1.23	.131
SMW––freedom at work	T1	50	4.79	1.02	32	4.13	1.35	.013
	T3	34	4.91	0.71	30	4.26	1.24	.015
SMW––learning and development	T1	50	4.99	1.02	32	4.55	1.35	.219
	T3	32	5.04	0.92	30	4.53	1.27	.098
SMW––quality of relations	T1	50	4.98	0.88	32	4.88	0.93	.633
	T3	32	5.24	0.67	30	4.98	0.71	.133
SMW–coherence and expressiveness at work	T1	48	4.30	0.99	31	4.48	1.43	.703
	T3	33	4.95	0.99	30	4.52	1.27	.262
WHOQOL––physical domain	T1	48	3.94	0.62	32	3.85	0.84	.573
	T3	33	3.93	0.56	30	3.89	0.69	.797
WHOQOL––psychological domain	T1	49	3.93	0.58	32	3.93	0.53	.904
	T3	33	3.92	0.58	30	4.08	0.50	.917
WHOQOL––social domain	T1	44	3.73	0.70	32	3.91	0.68	.269
	T3	32	3.76	0.63	30	3.99	0.65	.165
WHOQOL––environment domain	T1	47	3.67	0.57	32	3.65	0.52	.666
	T3	32	3.67	0.66	30	3.65	0.66	.788

^B^Minimal level of significance in the comparisons between groups; Student’s *t* test for independent groups

## Discussion

The objective of the present article was to evaluate the results obtained in a RPP carried out in a municipal public institution, regarding the expected outcomes: (a) changes in retirement planning behaviors, (b) changes in the meaning of work, and (c) quality-of-life improvement. The results were compared between a group of public workers who participated in a RPP and another group that did not, before, at the end, and 2 months after the program. Data on efficacy were analyzed considering times T1 and T2, T1 and T3, and T2 and T3.

Regarding the outcome change in retirement planning behaviors in the EG, there was a difference in the social-occupational investment, with an increase over time. Comparing with the CG, the same dimension was also higher for the EG in T3. Therefore, the data indicated that the program encouraged its participants to engage in activities other than work, raising interest in participation in community groups and investment in other projects that can be adapted/implemented after retirement, such as continuous improvement courses or voluntary work.

Concerning autonomy and well-being, which evaluates behavioral changes in daily life, such as the practice of physical activities, having a healthier diet, or having medical appointments and check-ups, no changes were observed among the participants in the program, although such themes have been worked on. This indicates the need to improve the way this content has been worked in the program. Furthermore, it is possible to assume that the time elapsed between the intervention and the follow-up may not have been sufficient to verify significant changes in the subjects’ behaviors regarding this factor.

In relation to the changes in the meaning of work, it was verified that the coherence and expressiveness factor at work stood out in the EG. According to the results, work seems to be meaningful for both the EG and the CG. However, all perceived the respect for human dignity, equality, valorization of justice, and preservation of rights in their work environment as weak, an aspect that requires attention in the discussions of future editions of the RPP.

On the other hand, freedom at work was different between the groups from T1, indicating that the EG presented more freedom to solve problems and make decisions. Since the meaning of work is a major aspect of people’s experience, its absence or frailty certainly impairs the performance of activities, insofar as they become only repetitive movements or purposeless actions because activities depend on the interpretation of the reason for their realization (purpose) and must be connected to the self-representation of people themselves, that is, to their identity (Bendassolli and Borges-Andrade 2015). In this sense, it can be inferred that for the CG, the participation in the RPP, which is a long-term activity, would involve having greater freedom and autonomy at work, characteristics that were not being perceived in their work environment, which may have hampered their participation.

In relation to the quality-of-life improvement, the only significant difference occurred in the environmental domain, with the mean in T2 significantly higher than the means in T1 and T3. The environment domain measures physical security and protection, home environment, financial resources, health and social care, opportunity to learn new information and skills, participation in recreation/leisure activities, and physical environment (pollution/noise/traffic/climate), aspects that are addressed in the program. This reveals the need to emphasize this theme in the RPP so that the changes produced will remain over time.

Regarding the sociodemographic profile, it is noteworthy that the female gender and the diversity of positions stand out. The fact that most participants are women corroborates the literature that indicates that women seek more information and care services related to their own health and that of the family (Leandro-França et al. 2015). There are also studies indicating that women plan retirement more than men and invest more in healthy behaviors, such as the practice of leisure activities and involvement in interpersonal relationships (Petkoska and Earl 2009). Leandro-França et al. (2015) emphasize that making retirement planning interventions that are more responsive to the male audience are rare. Thus, it becomes relevant to develop means to attract men to the RPPs or to make them more suitable to the demands and limitations of this group. Regarding positions, they ranged from operational to strategic level. The difference in relation to the labor activities influences different factors such as greater or lower recognition, workload, and stress in daily activities (Bressan et al. 2012), as well as greater adaptation to retirement, physical health decline, health problems in the family, financial shortages, and occupation and leisure activities (Leandro-França et al. 2014). Therefore, it is possible to think that such specificities may have reflected in the evaluated outcomes.

Evaluating the efficacy of the RPP, it was verified that in relation to the occupational investment, coherence and expressivity at work, and quality related to the environment, the EG presented important gains. There was also an improvement in the perception of health satisfaction, although the perception of quality of life declined in relation to the classifications good and very good, which may be because participants were faced with questions related to aging, which they had not considered yet.

Finally, the data presented in this research reinforce the need for a systematic and continuous evaluation of RPPs to provide important data in decision-making and to achieve effective results (Costa and Castanhar 2003). Hence, the importance of investing in actions that promote the quality of life of the citizens, since it reduces the possibilities of disease, leads to savings for public and private health services (França and Soares 2009).

## Conclusions

There is a consensus in the retirement literature that this process represents one of the most significant life-course transitions (Costa and Soares 2009; Duarte and Melo-Silva 2009; França 2009; Soares et al. 2007). Therefore, it is essential that organizations propose Retirement Preparation Programs that are validated empirically. In this sense, the present study contributed with the accomplishment of a quantitative evaluation with an experimental group and a three-time control group, considering that the national literature has presented qualitative evaluations without follow-up (Murta et al. 2008; Soares and Costa 2011; Zanelli et al. 2010).

It is important to emphasize the scientific and social relevance of investigating retirement planning behaviors, the meaning of work, and the quality of life in a group of public workers in the last stage of their careers, in order to propose actions and policies in the area of health, seeking to meet the demands of the aging population. Psychology becomes especially important within this context, as it can help people gain a better understanding of the moment they are experiencing to assist them in this preparation and transition by promoting healthy habits.

Some limitations, other than those already mentioned, may have affected the results obtained, such as the loss of participants throughout the study. This fact is predictable in longitudinal studies, considering the cost of participation and the changes that may occur over time (Kazdin 2010). In the EG, there were justified withdrawals due to the request to leave earlier because of other commitments (*n* = 5), work leave due to vacations (*n* = 2), work commitments (*n* = 1), and retirement itself. In the follow-up, the loss was even more important due to the 17 withdrawals (10 due to vacations or premium leave, 1 due to work commitment, and another due to retirement). The repetition of instruments at different moments may also have affected the results since there may be a memory effect, inducing participants to mark the same items at different moments of evaluation (Mednick et al. 1984). Another factor that may have affected the results was the time available to respond to the instruments (30 min) due to the scheduling of the program. Finally, it is possible that the disinterest in preparing for retirement moderated the change in relation to the control group, confused with the role of non-participation in the intervention.

As recommendations for new research agendas, it is suggested that future studies use an experimental design with a more robust sample, more equally stratified in relation to gender, and cover the different levels of education, income, and professional categories. Longitudinal studies are also recommended to follow participants after retirement, comparing those who participated in RPPs and those who did not participate in any preparatory activity. It is still important to compare the mortality rates between individuals who participated in RPPs and those who did not, as well as to analyze the prevalent causes, with the purpose of implementing actions to prevent the main diseases identified (Pinto, Schneider, Souza, & Seadi, 2016).

It is known that, while life expectancy increases, the number of benefits granted by the Brazilian Social Security System also increase and that this is a critical point for the government and the organizations. In Brazil, a social security reform is being discussed, whose objective is to maintain the sustainability of public accounts given the growing deficit of the social security system. Therefore, retirement is undoubtedly a current and important issue that will affect the lives of all Brazilian citizens. However, to ensure a decent life for citizens, it will be necessary to rethink politics, institutions, services, and practices as well as to prepare citizens in the short, medium, and long term for this new reality of retirement.

## Footnotes

1 Each year, four classes are offered, with 30 vacancies each; however, due to the cost containment, this year, only two classes were offered.

## Authors’ information

Tanise Amália Pazzim

Psychologist, graduated from Universidade Luterana do Brasil - ULBRA (2004), with a background in Organizational Psychology at the Global Development Institute - IDG (2005) and in Group Dynamics Coordination at the Brazilian Society of Group Dynamics - SBDG (2007). She is a specialist in Public Management by the Federal University of Rio Grande do Sul - UFRGS (2009) and Master in Clinical Psychology by the University of Vale do Rio dos Sinos - UNISINOS (2015/2016).

Angela Helena Marin

Psychologist, graduated from the Federal University of Santa Maria (2002) and holds a degree in psychology from the Federal University of Rio Grande do Sul (2007). She has a specialization in clinical psychology from the Porto Alegre Family Institute (2008), a master’s degree (2004) and a doctorate (2009) in Psychology from the Federal University of Rio Grande do Sul. Currently, she is a clinical psychologist and teacher of undergraduate and post-graduate degree in psychology from the University of Vale do Rio dos Sinos. She has experience in Psychology with emphasis in clinical psychology and human and family development, working mainly on the following topics: social development in childhood and adolescence, family and processes of prevention and health promotion.
