# Correction to: Retirement preparation program: evaluation of results

**DOI:** 10.1186/s41155-018-0091-2

**Published:** 2018-04-09

**Authors:** Psicologia: Reflexão e CríticaPsychology: Research and Review

**Affiliations:** London, UK

## Correction

The original publication (Psicologia: Reflexão e CríticaPsychology: Research and Review, 2017) contains an error in the article title. The original article was updated to rectify this error. The correct version can be found below:

- Incorrect version:
  - Retirement preparation program: evaluation of results
    Psychology: Research and Review
- It should read:
  - Retirement preparation program: evaluation of results
